# Impaired Cross-Talk between Mesolimbic Food Reward Processing and Metabolic Signaling Predicts Body Mass Index

**DOI:** 10.3389/fnbeh.2014.00359

**Published:** 2014-10-17

**Authors:** Joe J. Simon, Mandy Skunde, Maria Hamze Sinno, Timo Brockmeyer, Sabine C. Herpertz, Martin Bendszus, Wolfgang Herzog, Hans-Christoph Friederich

**Affiliations:** ^1^Department of General Internal Medicine and Psychosomatics, Centre for Psychosocial Medicine, University Hospital Heidelberg, Heidelberg, Germany; ^2^Department of General Adult Psychiatry, Centre for Psychosocial Medicine, University Hospital Heidelberg, Heidelberg, Germany; ^3^Department of Diagnostic and Interventional Radiology, University Hospital Heidelberg, Heidelberg, Germany

**Keywords:** food reward, obesity, ventral striatum, fMRI, leptin, insulin resistance

## Abstract

The anticipation of the pleasure derived from food intake drives the motivation to eat, and hence facilitate overconsumption of food, which ultimately results in obesity. Brain imaging studies provide evidence that mesolimbic brain regions underlie both general as well as food-related anticipatory reward processing. In light of this knowledge, the present study examined the neural responsiveness of the ventral striatum (VS) in participants with a broad BMI spectrum. The study differentiated between general (i.e., monetary) and food-related anticipatory reward processing. We recruited a sample of volunteers with greatly varying body weights, ranging from a low BMI (below 20 kg/m^2^) over a normal (20–25 kg/m^2^) and overweight (25–30 kg/m^2^) BMI, to class I (30–35 kg/m^2^) and class II (35–40 kg/m^2^) obesity. A total of 24 participants underwent functional magnetic resonance imaging while performing both a food and monetary incentive delay task, which allows to measure neural activation during the anticipation of rewards. After the presentation of a cue indicating the amount of food or money to be won, participants had to react correctly in order to earn “snack points” or “money coins,” which could then be exchanged for real food or money, respectively, at the end of the experiment. During the anticipation of both types of rewards, participants displayed activity in the VS, a region that plays a pivotal role in the anticipation of rewards. Additionally, we observed that specifically anticipatory food reward processing predicted the individual BMI (current and maximum lifetime). This relation was found to be mediated by impaired hormonal satiety signaling, i.e., increased leptin levels and insulin resistance. These findings suggest that heightened food reward motivation contributes to obesity through impaired metabolic signaling.

## Introduction

Obesity has become a global public health and socioeconomic problem (Swinburn et al., 2011), and has been related to higher all-cause mortality (Flegal et al., 2013). A number of environmental and social factors, nowadays, contribute to the increasing prevalence of obesity, such as heightened availability and accessibility of high caloric food, sedentary lifestyle, and unhealthy eating habits. However, the risk to become overweight or obese is also largely determined by biological and genetic factors that predispose individuals to the overconsumption of food (Hill and Peters, 1998; Mitchell et al., 2011).

Previous studies consistently found overweight and obesity to be related to alterations in neural food reward processing (Burger and Stice, 2011). There is evidence for both a hyper-responsivity as well as a hypo-responsivity of reward regions to food in overweight and obesity. Thus, it is a matter of debate whether obese people are overeating due to greater reward sensitivity or to compensate for a reward deficit. However, these theories do not dissociate between anticipatory and consummatory food reward, furthermore, the relation between initial vulnerability and brain adaptations to repeated overeating still remains unknown. Animal research has shown that the rewarding value of food shifts from food intake to the anticipation of food after conditioning to a food indicating cue has taken place (Schultz et al., 1993). Therefore, in accordance to the incentive-sensitization theory of addiction (Robinson and Berridge, 2000), repeated overeating may lead to a greater incentive salience and motivation for food intake (“wanting”) but less activation of reward regions to food receipt (“liking”). Correspondingly, previous studies have shown that heightened anticipatory food reward was related to excessive caloric intake (O’Doherty et al., 2002; Davis et al., 2004).

The anticipation of food intake as well as the processing of pictures of palatable food has been related to the activation of the mesolimbic reward system in previous studies (O’Doherty et al., 2002; Beaver et al., 2006). The ventral striatum (VS) is a key region in the processing of the incentive value and has been specifically associated with the anticipation of a pleasurable outcome (Knutson et al., 2001). Accordingly, activation in the VS has been related to anticipatory food reward processing (Siep et al., 2012) and has been shown to be positively related to the amount of food consumption (Lawrence et al., 2012). Furthermore, the level of activation of the VS predicted the success of a weight-loss program (Murdaugh et al., 2012) and showed a lower decrease in response to food consumption in obese compared to normal-weight adolescents (Bruce et al., 2010). These results support the assumption that overfeeding behavior could be due to an abnormally enhanced motivational response to food stimuli in obese individuals (Davis et al., 2004). However, further research is needed to examine whether incentive salience in obese patients is food specific or extends to general (i.e., monetary) rewards.

Hence, advances in brain imaging techniques and methods have provided new insights in mesolimbic reward circuit functions related to obesity. However, the interaction between these mesolimbic reward circuits and energy metabolism in obesity is largely unknown (Morton et al., 2006; Berthoud, 2011). Among the peripherally synthesized hormones that regulate food intake, insulin, and leptin serve as dominant adiposity and anorectic signals. These hormones interact with the hypothalamus but also directly with mesolimbic circuits to modulate reward and motivational aspects of food intake (Farooqi et al., 2007; Heni et al., 2012). Previous studies indicate that the physiological functions of leptin and insulin are to diminish the sensitivity of mesolimbic dopamine pathways, and thus decrease the incentive salience of rewards (Hommel et al., 2006; Figlewicz and Benoit, 2009). It is well known that hyperinsulinemia and insulin resistance coexist with obesity. Due to an insulin resistance of muscle and fat cells, obese patients need to up-regulate their insulin levels to control plasma glucose levels and to facilitate glucose uptake in these cells. However, causal links between insulin resistance and obesity are complex (Kahn et al., 2006). Furthermore, leptin levels are considered a crucial signal from adipose tissue to the brain providing information about long-term energy stores (Considine et al., 1996). Leptin replacement in leptin deficient human beings resulted in a reduction of food intake and decreased brain activation in the VS (Farooqi et al., 2007; Aotani et al., 2012).

Due to the common observation of increased leptin values and insulin resistance in obese patients, we hypothesize that these changes are linked to alterations in mesolimbic circuits during anticipatory food reward processing. There is preliminary evidence that the dopamine reducing effect of leptin in the mesolimbic reward system is impaired in obese patients (Grosshans et al., 2012). Furthermore, it has been found that insulin resistance correlated positively with mesolimbic activity in obese but not in normal-weight individuals (Kullmann et al., 2012; Jastreboff et al., 2013).

In order to be able to give a precise account of the relation between individual BMI, metabolic factors, and mesolimbic neurocircuit functioning, we recruited healthy participants with varying BMI levels. A stratified sampling procedure was employed to assure that different BMI levels were adequately represented, resulting in a sample of participants characterized by a broad distribution of BMI levels. We employed both a monetary and food incentive delay (FID) task to measure reward-related processing during the anticipation of rewards. Due to the importance of the VS in anticipatory reward processing, we focused our analyses on this region using a region of interest (ROI) approach.

The aim of the study was to investigate whether present BMI and maximum lifetime BMI are related to the responsiveness of the VS. Furthermore, we hypothesized that the linkage between the responsiveness of the VS and BMI is specific to food reward and independent of general reward anticipation (i.e., monetary reward). Finally, we hypothesized that metabolic satiety parameters such as leptin and insulin resistance exert a mediating effect on this relation, indicating an impaired interaction between homeostatic and mesolimbic reward pathways and increasing BMI.

## Materials and Methods

### Participants

Twenty-four healthy participants with different BMI levels took part in the study. To ensure a wide range of body weight in our sample, four participants were included with a BMI below 20 kg/m^2^ (low BMI) and five participants each with a BMI between 20 and 25 kg/m^2^ (normal BMI), between 25 and 30 kg/m^2^ (overweight), between 30 and 35 kg/m^2^ (class I obesity), and between 35 and 40 kg/m^2^ (class II obesity). Furthermore, participants in these subgroups were matched for age and education (Table 1). All participants had normal or corrected to normal vision, were right-handed and were screened for medical and mental diseases by taking the medical history, measuring body weight and height, and by interviewing them with the Structured Clinical Interview for DSM-IV (SCID, Wittchen et al., 1997). Exclusion criteria were metallic implants, claustrophobia, chronic illness, regular smoking, and a lifetime diagnoses of a mental disorder. To exclude participants with a diabetic metabolic status, only participants with a fasting glucose below 110 mg/dl were included. The study was approved by the Ethics Committee of the University of Heidelberg. All participants gave their written informed consent before entering the study, and received financial compensation for their participation in the study.

**Table 1:** **Participants demographics**.

Characteristics	All participants	Relation with BMI
	*M* (SD, range)	*r*
*N* total/*n* women	24/19	
Age (years)	28.6 (3.6; 24–34)	*r* = 0.001, *p* = 0.995
Education (years)	12.8 (1.7; 9–17)	*r*_s_ = −0.201, *p* = 0.345
BMI (kg/m^2^)	28.2 (7; 18.4–40)	
Glucose, fasting (mg/dl)	80.8 (17.5; 55–95)	*r*_s_ = 0.222, *p* = 0.309
Insulin, fasting (μU/ml)	7.3 (6.2; 1.7–23.5)	*r* = 0.356, *p* = 0.113
HOMA-IR	1.6 (1.3;0.17–4.8)	*r* = 0.388, *p* = 0.091
Leptin (ng/ml)	8.7 (6.3;0.83–21)	*r* = *0.706, p* < *0.001*
Maximum lifetime BMI	29.9 (7.2; 19.2–40.6)	*r* = *0.973, r* < *0.001*
FID snack points won	275.5 (55.3; 238–300)	*r*_s_ = −0.255, *p* = 0.230
MID money won	28.7 (1.7; 25–30)	*r*_s_ = −0.311, *p* = 0.139

*r, Pearson’s correlation coefficient; *r*_s_, Spearman’s rho; FID, food incentive delay task; MID, money incentive delay task. Significant correlations between BMI and the respective values are given in italic*.

### Study procedure

All participants were asked to come to the clinic without having breakfast and to refrain from smoking and consuming alcoholic drinks for 24 h before the experiment. Blood samples were collected immediately at the beginning of the study. Participants then received a light standardized breakfast at 9.00 a.m., followed by the SCID. After the interview, all participants were asked to conduct a battery of neuropsychological tasks and to complete a package of questionnaires. The findings of the former are not reported in this study. The MRI scanning was performed for all participants at 12:00 p.m., corresponding to the lunchtime of most of the participants.

### Biochemical evaluation

Blood samples were taken in the morning (8.30 a.m.) at the day of the fMRI measurement after an overnight fast, starting at 8.00 p.m. the evening before. After the blood was centrifuged under cold conditions, the serum was separated and stored at −80°C. Insulin and leptin were measured using commercial kits based on a sandwich ELISA assay from Merck Millipore (Merck KGA, Darmstadt, Germany) with a detection limit of 0.85 μU/ml and 0.2 ng/ml, respectively. The cross reactivity with related peptides, such as human C-peptide and human pro-insulin were not detectable at concentrations of 20 and 2 ng/ml with the insulin test. Each sample was measured in duplicate. The intra-assay coefficients of variation for insulin were 2.58 and 2.19% at concentrations of 7.75 and 45.63 μU/ml, respectively. The inter-assay coefficients of variations at these concentrations were below 4 and 12%, respectively. For leptin, the intra-assay coefficients of variations were 0.17 and 3.16% at concentrations of 2.52 and 15.82 ng/ml, respectively. The inter-assay coefficients of variations at these concentrations were below 21 and 10%, respectively. Glucose concentrations were performed at the central laboratory of the University of Heidelberg on a Siemens Advia 2400 device (Eschborn, Germany) using the hexokinase method. Insulin resistance was assessed by homeostasis model assessment of insulin resistance (HOMA-IR, calculated as follows: [glucose (mg/dl) × insulin (mU/ml)]/405) (Jastreboff et al., 2013). Due to technical reasons, the insulin values could not be evaluated for three participants and the glucose values could not be measured for one participant, leaving 20 participants for the final HOMA-IR analysis.

### Stimuli and task

**Figure 1:**
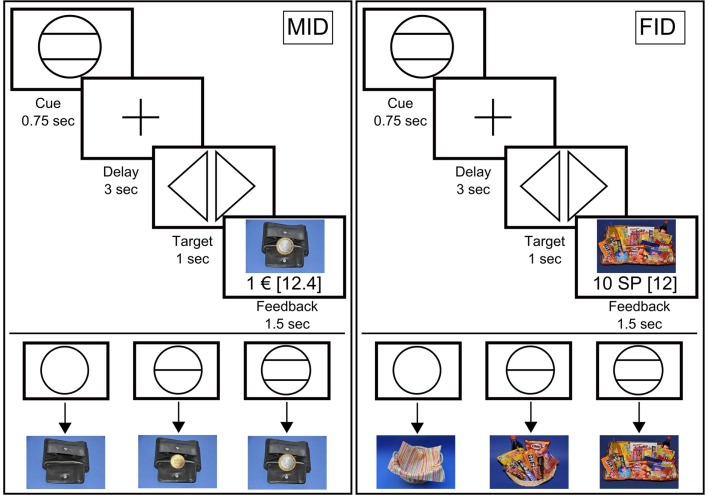
**Graphical depiction of the money (MID task) and food incentive delay task (FID task)**. The figure shows the cues that represent possible reward outcomes (EUR 1, cents 20 and EUR 0/10 snack points (SP), 2 SP and 0 SP, respectively) and the structure of the MID/FID task. Participants were presented with a cue stipulating the amount of money/SP that they could win if they reacted correctly during the following discrimination task. Immediately after target presentation, participants were informed about the amount of money/SP that they had won during the trial, including their cumulative total win by this point in time.

We used a modified version of the “monetary incentive delay” (MID) task as proposed by Abler and colleagues (Knutson et al., 2001; Abler et al., 2005). In a previous study, we showed that this paradigm allows an efficient probing of both anticipation and consumption of reward (Simon et al., 2010b). Additionally, we employed a “FID” task; instead of money, participants were able to win “snack points” (SP), which they then could use to buy sweet and salty snacks and beverages immediately after the MRI measurement. There were four blocks of reward tasks, consisting of 60 trials each. The block sequence was either SMSM (S = Snacks, M = Money), or MSMS, and was counterbalanced across participants. Before entering the scanner, participants performed a practice version of the task lasting 3 min for each condition for which they received neither payment nor snacks. The degree of potential rewards varied on three levels as indicated via graphical cues (Figure 1). In both tasks, each trial started with the presentation of a symbol (“cue,” 750 ms) indicating the amount of money/number of SP that they could win with a correct response (i.e., EUR 1, cents 20, EUR 0, or 10 SP, 2 SP, 0 SP, respectively). After an anticipation period (“delay”, 3000 ms), participants had to correctly react to one of two symbols (“targets”; i.e., triangle inclined to the right or a triangle inclined to the left) with a left or right button press corresponding to the direction of the triangle (index or middle finger of dominant hand) within a fixed interval of 1000 ms. This leads to a low task-difficulty with a very high success rate, independent of the participants’ reaction speed. In order to guarantee a steady rate of reward vs. non-reward throughout all participants, we used a probabilistic reward pattern, i.e., reward was not paid out in 30 predefined trials (out of the 80 reward trials). Immediately after target presentation, feedback appeared (“feedback,” 1500 ms), notifying participants about the amount of money/SP that they had won and about their cumulative total win. In order to increase statistical efficiency, trials were separated by jittered intertrial intervals (ITIs) ranging from 1 to 8 s, with a mean of 3.5 s. The MID task used graphical depictions showing a wallet filled with the corresponding amount of money won during each trial. The FID task used pictures of either a large basket filled with snacks, a small basket filled with snacks, or an empty basket, depending on the amount of SP won. An incorrect button press resulted in zero payout. To ensure that participants paid attention and responded to every experimental condition, a penalty of −EUR1/−10 SP was applied if they missed to press one of the two buttons. In the MID task, participants were able to win a maximum of EUR 30. In the FID task, the maximum amount to be won was 300 SP, with any snack of the basket being 50 SP worth.

### fMRI acquisition

Images were collected using a 3-T Siemens Trio MRI scanner (Siemens Medical Solutions, Erlangen, Germany) equipped with a standard 32 channel head coil. The participants performed four functional runs lasting 9.3 min each with 280 volumes per run. In order to minimize susceptibility artifacts in the orbitofrontal cortex 30 oblique slices (interleaved acquisition) with a 10° angle relative to the AC-PC axis were acquired with 1 mm interslice gap, using a T2*-sensitive single-shot EPI sequence with following parameters: TR = 2000 ms, TE = 30 ms, resulting in an in-plane resolution of 3 mm × 3 mm × 4 mm, flip angle = 80°, field of view = 192 mm × 192 mm. Participants viewed visual stimuli on a projection screen via a mirror fixed to the head coil and responded with the right hand using a button box. High-resolution T1 MPRAGE anatomical images were acquired (192 slices, voxel size 1 mm × 1 mm × 1 mm, TR 1570 ms, TE 2.63 ms, 9° flip angle) for anatomical reference.

### fMRI data analysis

Functional MRI data were pre-processed and analyzed with SPM8 (Statistical Parametric Mapping, Wellcome Department of Cognitive Neurology, London, UK). To account for magnetic field equilibration, four volumes from the start of each functional run were excluded from analysis. Pre-processing of functional scans included slice time correction (reference to the first slice, using SPM8’s Fourier phase shift interpolation), within-participant registration and unwarping of time-series (to correct for motion artifacts), coregistration of the T1 image with the mean T2*-image, spatial normalization of both the functional and structural images to a standard T1 brain template (ICBM152, Montreal Neurological Institute, MNI), resulting in a voxel size of 3 mm^3^ for functional images and a voxel size of 1 mm^3^ for high-resolution anatomic images, and smoothing with an 8 mm full-width half-maximum isotropic Gaussian kernel. A 128-s high-pass filter was used to remove low-frequency noise and signal drift.

At the first level of analysis, pre-processed functional MRI data for both tasks were analyzed separately in the context of the general linear model (GLM) approach (Friston et al., 1994). Regressors were modeled separately for the three different anticipation phases (anticipation of EUR 1, cents 20, and EUR 0 for the MID task, anticipation of 10 SP, 2 SP, and 0 SP for the FID task) and the five different outcome phases (receipt of EUR 1, omission of EUR 1, receipt of cents 20, omission of cents 20, and receipt of EUR 0/neutral outcome for the MID task, receipt of 10 SP, omission of 10 SP, receipt of 2 SP, omission of 2 SP, and receipt of 0 SP/neutral outcome for the FID task) as explanatory variables convolved with the gamma-variate function described by Cohen (1997). Targets and error trials were included as additional regressors of no interest. Linear combinations of the estimated GLM parameters allow the assessment of changes in the BOLD responses of individual participants, contingent on the experimental condition. Individual contrast images corresponding to the effects of interest were subsequently constructed. For the analysis of reward anticipation, we contrasted the anticipation of a high reward (EUR 1 or 10 SP) with the anticipation of no reward (EUR 0 or 0 SP, anticipation_high). The analysis of the impact of a rewarding outcome is reported elsewhere. All contrasts were modeled separately for each task.

At the second level of analysis, the individual contrast images of all participants were included in a random-effects analysis, allowing population inference (Holmes and Friston, 1998). Due to the specificity of our *a priori* hypotheses, a ROI analysis using the specific contrasts of interests was carried out in order to identify reward-sensitive brain areas. Within-group activation was compared using a one-sample *t*-test. We used an anatomical voxel-mask for the bilateral VS taken from a publication-based probabilistic MNI atlas (Nielsen and Hansen, 2002) as in previous studies (Schlagenhauf et al., 2008; Simon et al., 2010a). We report small-volume corrected results significant on a family wise error extent threshold of *p* < 0.05, and cluster defining threshold *p* < 0.001, uncorrected. The location of the peak activity associated with each cluster of activation is reported in MNI-coordinates. Based on the clusters of activation found in the structural VS ROIs, we then constructed functional ROIs (for both the left and right VS, respectively) in order to extract mean percent signal change using MarsBaR (Brett et al., 2002). The analysis of the impact of a rewarding outcome showed no significant differences within the mesolimbic system and was not considered for the further analyses. Linear regression analyzes were performed to quantify the influence of brain activation on BMI using SPSS version 20 (IBM Corp.; Armonk, NY, USA). The relation between the participants’ individual BMI and demographic values, blood parameters was assessed using Pearson’s *r* and Spearman’s rank-order correlation, respectively.

### Mediator analyses

To examine the hypothesized associative chain linking VS activity, HOMA-IR, leptin, and BMI, a serial multiple-mediator model was tested using the product-of-coefficients approach (MacKinnon et al., 2007). This method is superior to the more commonly used causal steps approach (Baron and Kenny, 1986) due to its higher power and its direct quantification of the intervening effects (MacKinnon et al., 2007; Hayes, 2009). Generally, mediator analyses are based on the assumption that the total effect of a predictor on an outcome (i.e., weight *c*) is composed of the direct effect of the predictor on the outcome that is independent of any mediator (i.e., weight *c*′), and the indirect effect of a predictor on an outcome through one or more mediators. An indirect effect refers to the product of the effect of the predictor on a mediator (i.e., weight *a*) and the effect of the mediator on the outcome (i.e., weight *b*). In the kind of mediator models that we have applied, the direct and indirect effects of a predictor are estimated using the coefficients from three equations (one for each mediator, *M*, and one for the outcome variable, *Y* – see also Figure 4):

$$\begin{array}{llll} & M_{1}=i_{M_{1}}+a_{1}X+e_{M_{1}}\; & & \;\\ & M_{2}=i_{M_{2}}+a_{2}X+a_{3}M_{1}+e_{M_{2}}\; & & \;\\ & Y=i_{Y}+c\prime_{1}X+b_{1}M_{1}+b_{2}M_{2}+e_{Y}\; & & \; \end{array}$$

HOMA-IR and leptin were included as serial mediators of the link between VS activity (predictor) and BMI (outcome). Within the mediator model, three specific indirect effects were tested (see also Figure 4). The first carries the effect of VS activity on BMI through HOMA-IR only (*a*_1_*b*_1_). The second carries the effect of VS activity on BMI through leptin only (*a*_2_*b*_2_). The third indirect effect carries the effect of VS activity on BMI through both HOMA-IR and leptin (*a*_1_*a*_3_*b*_2_). Adding these three indirect effects to the direct effect yields the total effect (*c*_1_). A Sobel test (Sobel, 1982) as well as bias-corrected and accelerated 95% bootstrap confidence intervals (Preacher and Hayes, 2008), based on 10,000 resamples, were used to assess the statistical significance of the indirect effects. Point estimates of indirect effects were considered significant if the confidence interval did not contain zero. All analyses were carried out using the PROCESS script for SPSS Statistics 20 developed by Hayes (2013).

## Results

In an initial correlation analysis, we found the participants’ individual BMI levels to be positively related with leptin values, and maximum lifetime BMI (Table 1).

### fMRI results

**Figure 2:**
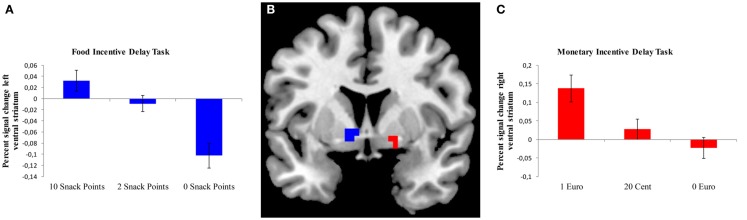
**Brain activation in the ventral striatum during the expectation of a food or monetary reward**. **(A)** Bar chart depicting percent signal change from the left ventral striatum (VS) during different anticipation conditions for the FID task. Error bars depict standard error of the mean. **(B)** ROI-masks were used to extract percent signal change for the FID task (left VS, depicted in blue) and MID task (right VS, depicted in red), rendered on a T1-weighted template image (coronal slice, *y* coordinate = 5 mm) supplied with micron (Colin brain). **(C)** Bar chart depicting percent signal change from the right VS during different anticipation conditions for the MID task. Error bars depict standard error of the mean.

We analyzed brain activation during the expectation of both food- and monetary-related reward compared to the expectation of no reward using a ROI approach for the VS (Table 2). During the anticipation of a high vs. no food-related reward, a cluster of activation was observed in the left and right VS; however, the cluster in the right VS only extended to three voxels. During the expectation of a high vs. no monetary reward, a cluster of activation was observed in the right VS (Figure 2).

**Table 2:** **Significant clusters of group activation (*n* = 24) during the expectation of food or monetary reward**.

Region	Anticipation of high vs. no reward				
	*t*	MNI coordinates			Active voxels
		*x*	*y*	*z*	
**Food-related reward**				
Left ventral striatum	4.07	−9	5	−6	9
Right ventral striatum	3.82	12	5	−6	3
**Monetary related reward**				
Left ventral striatum	X				
Right ventral striatum	3.78	18	5	−10	9

*Coordinates (*x, y, z*) reflect MNI space. Activations are small-volume corrected significant on a family wise error extent thresholds of *p* < 0.05, cluster defining threshold *p* < 0.001 uncorrected*.

### Correlation analyses and mediator effects

**Figure 3:**
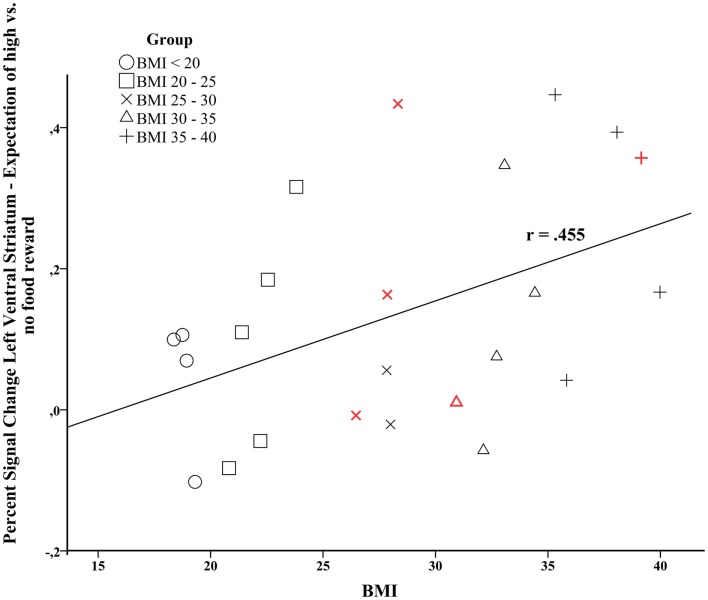
**Correlation analysis between brain activation in the left ventral striatum during the expectation of high vs. no food-related reward and individual BMI (Pearson’s *r* = 0.455, *p* = 0.026)**. Data points colored in red depict male participants.

The signal change in the left VS during the expectation of a high vs. no food reward was positively correlated with individual BMI levels (*r* = 0.455, *p* = 0.026, Figure 3). Furthermore, activity in the left VS was related to maximum lifetime BMI (*r* = 0.410, *p* = 0.047). We only observed a trend for a positive relation between activity in the right VS and current BMI (*r* = 0.352, *p* = 0.092).

To analyze the specificity of the observed correlations, we performed a correlation between activation in the right VS during the MID task and individual BMI levels to examine whether the link between neural reward processing and BMI is specific to food-related reward or also valid for general (i.e., monetary) reward. We observed neither a significant effect for current BMI (*r* = 0.202, *p* = 0.345) nor for maximum lifetime BMI (*r* = 0.244, *p* = 0.251).

**Figure 4:**
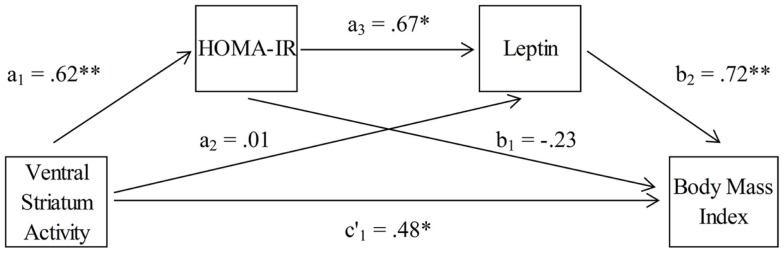
**Association between ventral striatum activation and body mass index, mediated by insulin resistance (HOMA-IR) and leptin (*n* = 18)**.

Finally, we tested whether leptin and insulin resistance (HOMA-IR) mediated the association between activity in the left and right VS during the FID task and BMI. We employed a serial multiple-mediator model with leptin and insulin resistance values as serial mediators of the association between activity in the VS and individual BMI levels (Figure 4).

To control for possible outliers in our data, we first calculated Cook’s distance (Cook’s D) to identify data points with large residuals (Cook and Weisberg, 1982), which were then excluded from the mediator analysis. We found the HOMA-IR value of one participant to be above the cut-off value [*D* = 1.09, accepted cut-off value: *D* > 4/N, where N is the number of observations, resulting in a value of *D* > 0.2 for the HOMA-IR values (Bollen and Jackman, 1990)]. Similarly, the leptin value of another participant was above the cut-off value (*D* = 0.457).

The results of the mediation analyses are given in Table 3. The total effect of left VS activity on BMI was *c*_1_ = 21.96 (SE = 9.58), *p* < 0.05. Examination of the specific indirect effects indicates that HOMA-IR and leptin operated in serial to mediate this association between VS activity and BMI. The strength of the first indirect effect of left VS activity on BMI through HOMA-IR only was *a*_1_*b*_1_ = −6.45 (SE = 12.19) with a 95% bootstrap confidence interval of −36.54 to 9.18. The second indirect effect of left VS activity on BMI through leptin only was *a*_2_*b*_2_ = 0.41 (SE = 9.98) with a 95% bootstrap confidence interval of −18.54 to 22.33. Hence, the first two indirect effects were not significant. However, the third indirect effect of left VS activity on BMI through both HOMA-IR and leptin was *a*_1_*a*_3_*b*_2_ = 13.72 (SE = 9.79) with a 95% bootstrap confidence interval of 2.83–51.63. Thus, the third indirect effect including both HOMA-IR and leptin as serial mediators of the link between left VS activity and BMI was significant. Finally, the direct effect of left VS activity on BMI (without HOMA-IR and leptin) was c′_1_ = 14.28 (SE = 9.97), *p* = 0.17. This means that HOMA-IR and leptin in concert totally mediated the association between left VS activity and BMI levels.

**Table 3:** **Path coefficients for the serial multiple-mediator model (*n* = 18)**.

	Path coefficient								
	To HOMA-IR			To leptin			To BMI		
	*b*	SE	β	*b*	SE	β	*b*	SE	β
Left VS	4.54	1.38	0.62**	0.50	9.63	0.01	14.28	9.97	0.31
HOMA-IR				3.67	1.35	0.67*	−1.42	1.7	−0.23
Leptin							0.82	0.27	0.72**
Total							21.96	9.58	0.48*

*With 10,000 bootstrap samples*.

*VS, ventral striatum; HOMA-IR, homeostasis model assessment of insulin resistance*.

*p < 0.05; **p < 0.01.

Similar results were found when performing the analysis for the right VS (data not shown). There was no significant effect of age as a covariate. However, when excluding male participants from the correlational analysis (*N* = 19), signal change in the left VS during the expectation of a high vs. no food reward correlated only trend-wise with individual BMI levels (*r* = 0.445, *p* = 0.056) as well as with maximum lifetime BMI (*r* = 0.409, *p* = 0.082). Similarly, including gender as a covariate in the mediator model rendered the indirect effect slightly non-significant (left VS activity on BMI through both HOMA-IR and leptin: 95% bootstrap confidence interval of −0.97 to 49.34).

## Discussion

The present study investigated the relation between neural processing of anticipatory food reward, BMI, and metabolic signaling in a sample of healthy individuals with a broad BMI spectrum. With respect to our first hypothesis, we confirmed that increased neural activation during the anticipation of food reward was related to increased current and maximum lifetime body weight. This observation was specific to food reward processing, as we observed no relation between BMI and monetary reward processing, confirming our second hypothesis. Our third hypothesis was also approved, in that the relationship between neural food-related reward processing and body weight was mediated by insulin resistance and leptin.

The incentive-sensitization theory of addiction (Robinson and Berridge, 2000, 2008) postulates that recurrent drug abuse induces a sensitization of mesolimbic reward networks, causing abnormal incentive motivation for drugs. Correspondingly, it has been discussed if sensitization to food could also be an underlying cause for obesity (Davis et al., 2004; Berthoud et al., 2011; Temple and Epstein, 2012). In line with previous observations (Lawrence et al., 2012; Murdaugh et al., 2012), our results indicate that an increased neural reward processing during the anticipation (“wanting”) of food-related rewards is associated with higher current and maximum lifetime BMI, which is in line with the notion that increased reward processing of food stimuli may represent a vulnerability marker for obesity. Importantly, we observed this relation for food related, but not for monetary related stimuli, arguing against the assumption that obesity results from a general hyperactive mesolimbic reward system.

Aberrant functioning of mesolimbic reward networks is thought to induce pathological motivation by hijacking associative learning processes (Robinson and Berridge, 2008), creating enhanced conditioned responses to specific reward predicting cues. Besides the VS that was in the focus of the present study, an emerging view in the literature is that the dorsal portion of the striatum (DS) plays an important part in compulsive eating habits (Everitt and Robbins, 2013). Critical in the selection of actions on the basis of their currently expected reward value (Balleine et al., 2007), increased activity in the DS, and a concurrent decrease in brain regions subserving inhibitory control (mainly the dorsolateral prefrontal cortex) have been found in obese individuals during anticipatory food reward (Rothemund et al., 2007; Nummenmaa et al., 2012). Furthermore, during consummatory food reward, adolescents at risk for obesity showed increased activity in the DS (Stice et al., 2011), whereas obese adults showed decreased activity in the DS (Stice et al., 2008a,b). Since we found no significant neural activation within the mesolimbic system during the receipt of reward in this particular sample, we were not able to conduct a similar correlation and mediation analysis on brain activation, metabolic signaling, and BMI level for the liking component of food reward processing. However, taken together with previous studies, these results may indicate that during the anticipation of food reward (i.e., “wanting”), both ventral and dorsal striatal regions are hyper-activated in obese participants, creating an increased sensitization as well as biased action selection toward food-related stimuli, whereas the consumption of food (i.e., “liking”) is accompanied by reduced dorsal striatal activity in obese individuals, which may cause obese individuals to overeat in order to compensate for this reward deficiency (Burger and Stice, 2011). However, numerous other brain regions are involved in food reward processing including the extended central amygdala (i.e., bed nucleus of the stria terminalis, BNST). In animal research, it was shown that midbrain leptin receptor neurons also show close connections to the extended central amygdala (Opland et al., 2010). As the VS and the BNST are anatomically in close neighborhood, the resolution of the applied fMRI protocol may have not been sufficient to clearly distinguish between these two regions. Therefore, high-resolution fMRI should be used in the future to more clearly distinguish between these two regions.

In the present study, increased VS processing during the anticipation of food reward was mediated by both increased insulin resistance and leptin levels. Both leptin and insulin receptors are expressed within the mesolimbic system, specifically in the ventral tegmental area (Figlewicz et al., 2003). Physiologically, leptin and insulin provide an inhibitory, homeostatic feedback to the mesolimbic dopaminergic system, hence, preventing excessive intake of nutrients (Farooqi et al., 2007; Heni et al., 2012). Thus, the findings of a positive correlation between ventral striatal activity and the metabolic factors of leptin and insulin resistance indicate an impaired feedback mechanism. Our mediation model indicates that insulin resistance and leptin values operate in serial to mediate the relationship between VS activity and BMI. Serum leptin levels are higher in obese compared to lean participants with positive correlation with percentage of body fat (Considine et al., 1996), as adipocytes secrete leptin in direct proportion to adipose tissue mass. But leptin expression and secretion are also regulated by a variety of other factors. For example, leptin is increased by insulin (Kershaw and Flier, 2004). Experimental animal research and studies in human beings have shown that hyperinsulinemia increased plasma leptin concentrations (Saladin et al., 1995; Havel et al., 1998). Conclusively, insulin appears to act directly at the level of the adipocyte by increasing leptin secretion, perhaps due to increased glucose transport and metabolism (Margetic et al., 2002). This may be reflected by our serial mediation model, where hyperinsulinemia and insulin resistance acted as precursor for higher leptin values, which then together accounted for the relation between VS activity and BMI values. Therefore, changes in metabolic functioning in obese individuals may lead to a central resistance of the mesolimbic dopaminergic system, contributing to an aberrant neural processing of anticipatory food reward. The present findings corroborate previous studies that showed an impaired interaction between leptin and insulin and the mesolimbic reward circuitry in obesity (Grosshans et al., 2012; Jastreboff et al., 2013). Since our sample included a broad spectrum of BMI, ranging from underweight over normal weight and overweight to obesity, we were able to extend this relationship for normal and underweight individuals. However, due to the cross-sectional design of the present study, it still remains unclear whether this observation is related to a predisposition for obesity; further studies using a longitudinal approach are needed to assess the predictive value of this metabolic phenotype. There is preliminary evidence from longitudinal research indicating that greater striatal activity to food predicts future weight gain in normal-weight adolescents (Yokum et al., 2014). Therefore, it remains unclear whether ventral striatal responsivity is a cause or a consequence of overeating and obesity. In an effort to acknowledge the complexity of the issue of “who came first,” Carnell et al. (2012) propose a “Dynamic distributed neurobehavioral vulnerability model,” which postulates multifactorial forms of obesity associated with manifold phenotypes such as genetic, biological, and environmental factors. Accordingly, different routes giving differing factors higher weights may lead to obesity. Future studies should therefore investigate the conditions under which certain vulnerability factors become more relevant than others. A further limitation of the present study is the small sample size of the different BMI-groups. As each group was composed of only five participants, we were not able to assess differences between single groups. Further studies investigating these differences in larger samples are needed. Additionally, since we used only abstract rewards, the observed results should be replicated by adapting a more naturalistic approach, although the use of abstract rewards allows the analysis of food reward processing irrespective of individual food preferences. Furthermore, a number of additional metabolic factors such as ghrelin or Peptide YY, which we did not measure, should be included in future studies. Furthermore, both the effects in the correlational and mediator analyses were rendered marginally significant when taking gender into account. This may indicate a significant effect of gender on the observed associations. However, the medium effect sizes suggest that the non-significant results for the female-only subsample are rather due to the reduced power resulting from the narrowed sample size. Nevertheless, future studies investigating hormonal influences on the link between neural food reward processing and body weight should incorporate more balanced samples with respect to gender. Since our task was not designed to assess impulse control in our participants, we were not able to analyze cortical brain regions relevant for top down control. As impaired inhibitory control has been identified as a vulnerability marker for addiction and obesity (Verdejo-Garcia et al., 2008; Volkow et al., 2013), and has also been found to be related to the processing of food cues in the nucleus accumbens (Lawrence et al., 2012), future studies should address this issue.

This study demonstrates that ventral striatal activity in response to anticipatory food but not monetary reward predicts current and maximum lifetime body weight, supporting the incentive-salience model of obesity. This association is mediated by up-regulated metabolic factors that seem to have lost their inhibitory homeostatic feedback function on the mesolimbic reward system in obese individuals, causing overconsumption of high caloric food. Therefore, gaining further insights into the pathological cross-talk between metabolic factors and brain activation in the development and maintenance of obesity may facilitate the development of new obesity treatments.

## Conflict of Interest Statement

The authors declare that the research was conducted in the absence of any commercial or financial relationships that could be construed as a potential conflict of interest.

## References

[B1] Abler B. Walter H. Erk S. (2005). Neural correlates of frustration. Neuroreport 16, 669–672. 10.1097/00001756-200505120-00003 15858403

[B2] Aotani D. Ebihara K. Sawamoto N. Kusakabe T. Aizawa-Abe M. Kataoka S. (2012). Functional magnetic resonance imaging analysis of food-related brain activity in patients with lipodystrophy undergoing leptin replacement therapy. J. Clin. Endocrinol. Metab. 97, 3663–3671. 10.1210/jc.2012-1872 22872692 PMC3462942

[B3] Balleine B. W. Delgado M. R. Hikosaka O. (2007). The role of the dorsal striatum in reward and decision-making. J. Neurosci. 27, 8161–8165. 10.1523/JNEUROSCI.1554-07.2007 17670959 PMC6673072

[B4] Baron R. M. Kenny D. A. (1986). The moderator–mediator variable distinction in social psychological research: conceptual, strategic, and statistical considerations. J. Pers. Soc. Psychol. 51, 1173–1182. 10.1037/0022-3514.51.6.1173 3806354

[B5] Beaver J. D. Lawrence A. D. Van Ditzhuijzen J. Davis M. H. Woods A. Calder A. J. (2006). Individual differences in reward drive predict neural responses to images of food. J. Neurosci. 26, 5160–5166. 10.1523/JNEUROSCI.0350-06.2006 16687507 PMC6674259

[B6] Berthoud H. R. (2011). Metabolic and hedonic drives in the neural control of appetite: who is the boss? Curr. Opin. Neurobiol. 21, 888–896. 10.1016/j.conb.2011.09.004 21981809 PMC3254791

[B7] Berthoud H. R. Lenard N. R. Shin A. C. (2011). Food reward, hyperphagia, and obesity. Am. J. Physiol. Regul. Integr. Comp. Physiol. 300, R1266–R1277. 10.1152/ajpregu.00028.2011 21411768 PMC3119156

[B8] Bollen K. A. Jackman R. W. (1990). Regression diagnostics: an expository treatment of outliers and influential cases. Mod. Methods Data Analy. 13, 257–291

[B9] Brett M. Anton J. L. Valabregue R. Poline J. B. (2002). Region of interest analysis using the MarsBar toolbox for SPM 99. Neuroimage 16, S497

[B10] Bruce A. S. Holsen L. M. Chambers R. J. Martin L. E. Brooks W. M. Zarcone J. R. (2010). Obese children show hyperactivation to food pictures in brain networks linked to motivation, reward and cognitive control. Int. J. Obes. (Lond.) 34, 1494–1500. 10.1038/ijo.2010.84 20440296 PMC6800141

[B11] Burger K. S. Stice E. (2011). Variability in reward responsivity and obesity: evidence from brain imaging studies. Curr. Drug Abuse Rev. 4, 182–189. 10.2174/1874473711104030182 21999692 PMC3462740

[B12] Carnell S. Gibson C. Benson L. Ochner C. N. Geliebter A. (2012). Neuroimaging and obesity: current knowledge and future directions. Obes. Rev. 13, 43–56. 10.1111/j.1467-789X.2011.00927.x 21902800 PMC3241905

[B13] Cohen M. S. (1997). Parametric analysis of fMRI data using linear systems methods. Neuroimage 6, 93–103. 10.1006/nimg.1997.0278 9299383

[B14] Considine R. V. Sinha M. K. Heiman M. L. Kriauciunas A. Stephens T. W. Nyce M. R. (1996). Serum immunoreactive-leptin concentrations in normal-weight and obese humans. N. Engl. J. Med. 334, 292–295. 10.1056/NEJM199602013340503 8532024

[B15] Cook R. D. Weisberg S. (1982). Residuals and influence in regression. New York: Chapman and Hall

[B16] Davis C. Strachan S. Berkson M. (2004). Sensitivity to reward: implications for overeating and overweight. Appetite 42, 131–138. 10.1016/j.appet.2003.07.004 15010176

[B17] Everitt B. J. Robbins T. W. (2013). From the ventral to the dorsal striatum: devolving views of their roles in drug addiction. Neurosci. Biobehav. Rev. 37, 1946–1954. 10.1016/j.neubiorev.2013.02.010 23438892

[B18] Farooqi I. S. Bullmore E. Keogh J. Gillard J. O’Rahilly S. Fletcher P. C. (2007). Leptin regulates striatal regions and human eating behavior. Science 317, 1355. 10.1126/science.1144599 17690262 PMC3838941

[B19] Figlewicz D. P. Benoit S. C. (2009). Insulin, leptin, and food reward: update 2008. Am. J. Physiol. Regul. Integr. Comp. Physiol. 296, R9–R19. 10.1152/ajpregu.90725.2008 18945945 PMC2636975

[B20] Figlewicz D. P. Evans S. B. Murphy J. Hoen M. Baskin D. G. (2003). Expression of receptors for insulin and leptin in the ventral tegmental area/substantia nigra (VTA/SN) of the rat. Brain Res. 964, 107–115. 10.1016/S0006-8993(02)04087-8 12573518

[B21] Flegal K. M. Kit B. K. Orpana H. Graubard B. I. (2013). Association of all-cause mortality with overweight and obesity using standard body mass index categories: a systematic review and meta-analysis. JAMA 309, 71–82. 10.1001/jama.2012.113905 23280227 PMC4855514

[B22] Friston K. J. Holmes A. P. Worsley K. J. Poline J. P. Frith C. D. Frackowiak R. S. J. (1994). Statistical parametric maps in functional imaging: a general linear approach. Hum. Brain Mapp. 2, 189–210. 10.1002/hbm.460020402

[B23] Grosshans M. Vollmert C. Vollstadt-Klein S. Tost H. Leber S. Bach P. (2012). Association of leptin with food cue-induced activation in human reward pathways. Arch. Gen. Psychiatry 69, 529–537. 10.1001/archgenpsychiatry.2011.1586 22566584

[B24] Havel P. J. Uriu-Hare J. Y. Liu T. Stanhope K. L. Stern J. S. Keen C. L. (1998). Marked and rapid decreases of circulating leptin in streptozotocin diabetic rats: reversal by insulin. Am. J. Physiol. 274, R1482–R1491. 9612417 10.1152/ajpregu.1998.274.5.R1482

[B25] Hayes A. F. (2009). Beyond Baron and Kenny: statistical mediation analysis in the new millennium. Commun. Monogr. 76, 408–420. 10.1080/03637750903310360

[B26] Hayes A. F. (2013). An Introduction to Mediation, Moderation, and Conditional Process Analysis: A Regression-Based Approach. New York: Guilford Press

[B27] Heni M. Kullmann S. Ketterer C. Guthoff M. Linder K. Wagner R. (2012). Nasal insulin changes peripheral insulin sensitivity simultaneously with altered activity in homeostatic and reward-related human brain regions. Diabetologia 55, 1773–1782. 10.1007/s00125-012-2528-y 22434537

[B28] Hill J. O. Peters J. C. (1998). Environmental contributions to the obesity epidemic. Science 280, 1371–1374. 10.1126/science.280.5368.1371 9603719

[B29] Holmes A. P. Friston K. J. (1998). Generalisability, random effects & population inference. Neuroimage 7, S754

[B30] Hommel J. D. Trinko R. Sears R. M. Georgescu D. Liu Z. W. Gao X. B. (2006). Leptin receptor signaling in midbrain dopamine neurons regulates feeding. Neuron 51, 801–810. 10.1016/j.neuron.2006.08.023 16982424

[B31] Jastreboff A. M. Sinha R. Lacadie C. Small D. M. Sherwin R. S. Potenza M. N. (2013). Neural correlates of stress- and food cue-induced food craving in obesity: association with insulin levels. Diabetes Care 36, 394–402. 10.2337/dc12-1112 23069840 PMC3554293

[B32] Kahn S. E. Hull R. L. Utzschneider K. M. (2006). Mechanisms linking obesity to insulin resistance and type 2 diabetes. Nature 444, 840–846. 10.1038/nature05482 17167471

[B33] Kershaw E. E. Flier J. S. (2004). Adipose tissue as an endocrine organ. J. Clin. Endocrinol. Metab. 89, 2548–2556. 10.1210/jc.2004-0395 15181022

[B34] Knutson B. Adams C. M. Fong G. W. Hommer D. (2001). Anticipation of increasing monetary reward selectively recruits nucleus accumbens. J. Neurosci. 21, RC159. 11459880 10.1523/JNEUROSCI.21-16-j0002.2001 PMC6763187

[B35] Kullmann S. Heni M. Veit R. Ketterer C. Schick F. Haring H. U. (2012). The obese brain: association of body mass index and insulin sensitivity with resting state network functional connectivity. Hum. Brain Mapp. 33, 1052–1061. 10.1002/hbm.21268 21520345 PMC6870244

[B36] Lawrence N. S. Hinton E. C. Parkinson J. A. Lawrence A. D. (2012). Nucleus accumbens response to food cues predicts subsequent snack consumption in women and increased body mass index in those with reduced self-control. Neuroimage 63, 415–422. 10.1016/j.neuroimage.2012.06.070 22776461

[B37] MacKinnon D. P. Fairchild A. J. Fritz M. S. (2007). Mediation analysis. Annu. Rev. Psychol. 58, 593–614. 10.1146/annurev.psych.58.110405.085542 16968208 PMC2819368

[B38] Margetic S. Gazzola C. Pegg G. G. Hill R. A. (2002). Leptin: a review of its peripheral actions and interactions. Int. J. Obes. Relat. Metab. Disord. 26, 1407–1433. 10.1038/sj.ijo.0802142 12439643

[B39] Mitchell N. S. Catenacci V. A. Wyatt H. R. Hill J. O. (2011). Obesity: overview of an epidemic. Psychiatr. Clin. North Am. 34, 717–732. 10.1016/j.psc.2011.08.005 22098799 PMC3228640

[B40] Morton G. J. Cummings D. E. Baskin D. G. Barsh G. S. Schwartz M. W. (2006). Central nervous system control of food intake and body weight. Nature 443, 289–295. 10.1038/nature05026 16988703

[B41] Murdaugh D. L. Cox J. E. Cook E. W. III Weller R. E. (2012). fMRI reactivity to high-calorie food pictures predicts short- and long-term outcome in a weight-loss program. Neuroimage 59, 2709–2721. 10.1016/j.neuroimage.2011.10.071 22332246 PMC3287079

[B42] Nielsen F. Å. Hansen L. K. (2002). Automatic anatomical labeling of Talairach coordinates and generation of volumes of interest via the BrainMap database. Neuroimage 16(Suppl. 2).

[B43] Nummenmaa L. Hirvonen J. Hannukainen J. C. Immonen H. Lindroos M. M. Salminen P. (2012). Dorsal striatum and its limbic connectivity mediate abnormal anticipatory reward processing in obesity. PLoS ONE 7:e31089. 10.1371/journal.pone.0031089 22319604 PMC3272045

[B44] O’Doherty J. P. Deichmann R. Critchley H. D. Dolan R. J. (2002). Neural responses during anticipation of a primary taste reward. Neuron 33, 815–826. 10.1016/S0896-6273(02)00603-7 11879657

[B45] Opland D. M. Leinninger G. M. Myers M. G. Jr. (2010). Modulation of the mesolimbic dopamine system by leptin. Brain Res. 1350, 65–70. 10.1016/j.brainres.2010.04.028 20417193 PMC2921997

[B46] Preacher K. J. Hayes A. F. (2008). Asymptotic and resampling strategies for assessing and comparing indirect effects in multiple mediator models. Behav. Res. Methods 40, 879–891. 10.3758/BRM.40.3.879 18697684

[B47] Robinson T. E. Berridge K. C. (2000). The psychology and neurobiology of addiction: an incentive-sensitization view. Addiction 95(Suppl. 2), S91–S117. 10.1046/j.1360-0443.95.8s2.19.x 11002906

[B48] Robinson T. E. Berridge K. C. (2008). Review. The incentive sensitization theory of addiction: some current issues. Philos. Trans. R. Soc. Lond. B Biol. Sci. 363, 3137–3146. 10.1098/rstb.2008.0093 18640920 PMC2607325

[B49] Rothemund Y. Preuschhof C. Bohner G. Bauknecht H. C. Klingebiel R. Flor H. (2007). Differential activation of the dorsal striatum by high-calorie visual food stimuli in obese individuals. Neuroimage 37, 410–421. 10.1016/j.neuroimage.2007.05.008 17566768

[B50] Saladin R. De Vos P. Guerre-Millo M. Leturque A. Girard J. Staels B. (1995). Transient increase in obese gene expression after food intake or insulin administration. Nature 377, 527–529. 10.1038/377527a0 7566150

[B51] Schlagenhauf F. Juckel G. Koslowski M. Kahnt T. Knutson B. Dembler T. (2008). Reward system activation in schizophrenic patients switched from typical neuroleptics to olanzapine. Psychopharmacology (Berl.) 196, 673–684. 10.1007/s00213-007-1016-4 18097655

[B52] Schultz W. Apicella P. Ljungberg T. (1993). Responses of monkey dopamine neurons to reward and conditioned stimuli during successive steps of learning a delayed response task. J. Neurosci. 13, 900–913. 8441015 10.1523/JNEUROSCI.13-03-00900.1993 PMC6576600

[B53] Siep N. Roefs A. Roebroeck A. Havermans R. Bonte M. Jansen A. (2012). Fighting food temptations: the modulating effects of short-term cognitive reappraisal, suppression and up-regulation on mesocorticolimbic activity related to appetitive motivation. Neuroimage 60, 213–220. 10.1016/j.neuroimage.2011.12.067 22230946

[B54] Simon J. J. Biller A. Walther S. Roesch-Ely D. Stippich C. Weisbrod M. (2010a). Neural correlates of reward processing in schizophrenia – relationship to apathy and depression. Schizophr. Res. 118, 154–161. 10.1016/j.schres.2009.11.007 20005675

[B55] Simon J. J. Walther S. Fiebach C. J. Friederich H. C. Stippich C. Weisbrod M. (2010b). Neural reward processing is modulated by approach- and avoidance-related personality traits. Neuroimage 49, 1868–1874. 10.1016/j.neuroimage.2009.09.016 19770056

[B56] Sobel M. E. (1982). “Asymptotic confidence intervals for indirect effects in structural equation models,” in Sociological Methodology, ed. Leinhardt S. (San Francisco: Jossey-Bass), 290–312

[B57] Stice E. Spoor S. Bohon C. Small D. M. (2008a). Relation between obesity and blunted striatal response to food is moderated by TaqIA A1 allele. Science 322, 449–452. 10.1126/science.1161550 18927395 PMC2681095

[B58] Stice E. Spoor S. Bohon C. Veldhuizen M. G. Small D. M. (2008b). Relation of reward from food intake and anticipated food intake to obesity: a functional magnetic resonance imaging study. J. Abnorm. Psychol. 117, 924–935. 10.1037/a0013600 19025237 PMC2681092

[B59] Stice E. Yokum S. Burger K. S. Epstein L. H. Small D. M. (2011). Youth at risk for obesity show greater activation of striatal and somatosensory regions to food. J. Neurosci. 31, 4360–4366. 10.1523/JNEUROSCI.6604-10.2011 21430137 PMC3260083

[B60] Swinburn B. A. Sacks G. Hall K. D. Mcpherson K. Finegood D. T. Moodie M. L. (2011). The global obesity pandemic: shaped by global drivers and local environments. Lancet 378, 804–814. 10.1016/S0140-6736(11)60813-1 21872749

[B61] Temple J. L. Epstein L. H. (2012). Sensitization of food reinforcement is related to weight status and baseline food reinforcement. Int J Obes (Lond) 36, 1102–1107. 10.1038/ijo.2011.210 22041984

[B62] Verdejo-Garcia A. Lawrence A. J. Clark L. (2008). Impulsivity as a vulnerability marker for substance-use disorders: review of findings from high-risk research, problem gamblers and genetic association studies. Neurosci. Biobehav. Rev. 32, 777–810. 10.1016/j.neubiorev.2007.11.003 18295884

[B63] Volkow N. D. Wang G. J. Tomasi D. Baler R. D. (2013). The addictive dimensionality of obesity. Biol. Psychiatry 73, 811–818. 10.1016/j.biopsych.2012.12.020 23374642 PMC4827347

[B64] Wittchen H. Zaudig M. Fydrich T. (1997). Structured Clinical Interview for DSM-IV – German Version. Göttingen: Hoegrefe

[B65] Yokum S. Gearhardt A. N. Harris J. L. Brownell K. D. Stice E. (2014). Individual differences in striatum activity to food commercials predict weight gain in adolescents. Obesity (Silver Spring) 10.1002/oby.20882 25155745 PMC4236252

