# Corrigendum: Impaired cross-talk between mesolimbic food reward processing and metabolic signaling predicts body mass index

**DOI:** 10.3389/fnbeh.2014.00433

**Published:** 2014-12-12

**Authors:** Joe J. Simon, Mandy Skunde, Maria Hamze Sinno, Timo Brockmeyer, Sabine C. Herpertz, Martin Bendszus, Wolfgang Herzog, Hans-Christoph Friederich

**Affiliations:** ^1^Department of General Internal Medicine and Psychosomatics, Centre for Psychosocial Medicine, University Hospital HeidelbergHeidelberg, Germany; ^2^Department of General Adult Psychiatry, Centre for Psychosocial Medicine, University Hospital HeidelbergHeidelberg, Germany; ^3^Department of Diagnostic and Interventional Radiology, University Hospital HeidelbergHeidelberg, Germany

**Keywords:** food reward, obesity, ventral striatum, fMRI, leptin, insulin resistance

We have found a small error in Figure 4 in our manuscript. Inadvertently, we presented the beta value for the total effect instead of the beta value for the direct effect there. As also written in the text and in **Table 3**, under the arrow that connects “Ventral Striatum Activity” with “Body Mass Index” it must read c′1 = 0.31 (without asterisks). Please find attached the amended Figure.

**Figure 4:**
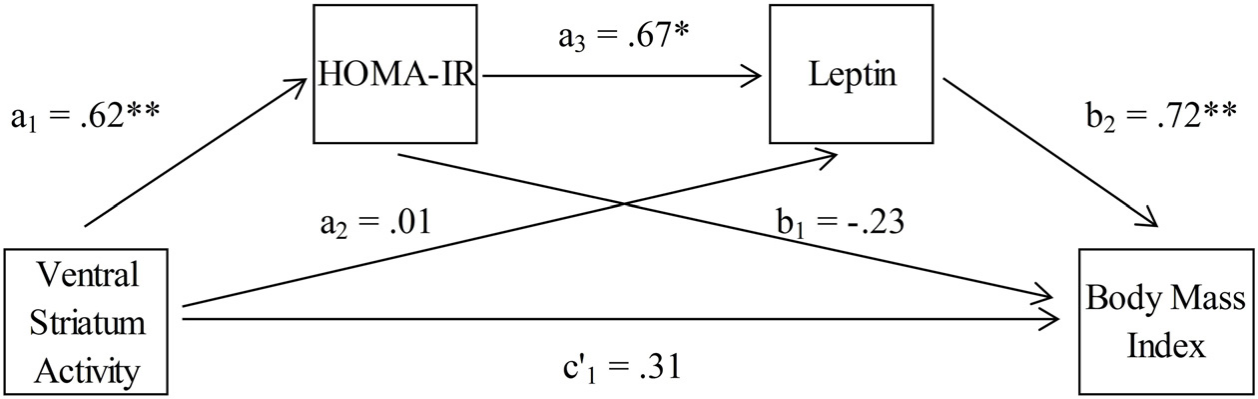
**Association between ventral striatum activation and body mass index, mediated by insulin resistance (HOMA-IR) and leptin (*n* = 18)**.

## Conflict of interest statement

The authors declare that the research was conducted in the absence of any commercial or financial relationships that could be construed as a potential conflict of interest.

